# What happens to young adults who have engaged in self-injurious behavior as adolescents? A 10-year follow-up

**DOI:** 10.1007/s00787-020-01533-4

**Published:** 2020-04-21

**Authors:** Daiva Daukantaitė, Lars-Gunnar Lundh, Margit Wångby-Lundh, Benjamin Claréus, Jonas Bjärehed, Ya Zhou, Sophie I. Liljedahl

**Affiliations:** 1 Department of Psychology, Lund University, Box 213, 221 00 Lund, Sweden; 2 Department of Clinical Sciences, Lund University, Lund, Sweden

**Keywords:** Adolescence, Deliberate self-harm, Non-suicidal self-injury, Young adulthood, Longitudinal

## Abstract

This study examined the longitudinal associations between non-suicidal self-injury (NSSI) in early adolescence and various positive and negative aspects of mental health in young adulthood. The participants were a cohort of regular school students (*n* = 1064) in grades 7–8 from a Swedish municipality. Nine hundred and ninety-one of these completed an 11-page questionnaire (T1: *M*_age_ = 13.7; 50.3% girls); 1 year later, 984 students completed the questionnaire again (T2: *M*_age_ = 14.8; 51.1% girls); and 10 years later, 557 took part (T3: *M*_age_ = 25.3; 59.2% women). The prevalence of any NSSI (≥ 1 instance) decreased from about 40% in adolescence to 18.7% in young adulthood, while the prevalence of repetitive NSSI (≥ 5 instances) decreased from about 18 to 10%. Compared to individuals who reported no NSSI as adolescents, and controlling for gender and psychological difficulties in adolescence, adolescents with stable repetitive NSSI (i.e., repetitive NSSI at both T1 and T2) showed significantly higher levels of stress, anxiety, NSSI, and difficulties in emotion regulation 10 years later. Even infrequent and unstable repetitive NSSI in adolescence was associated with negative outcomes in young adulthood. These results suggest that stable repetitive NSSI in adolescence is a strong risk factor for mental health problems in young adulthood and that occasional engagement in NSSI in adolescence is an indicator of vulnerability for poorer mental health in young adulthood.

**Electronic supplementary material:**

The online version of this article (10.1007/s00787-020-01533-4) contains supplementary material, which is available to authorized users.

## Introduction

Self-injurious behavior, either suicidal or non-suicidal, involves the deliberate infliction of harm on oneself [1]. Different terms have been used to describe such behavior. Deliberate self-harm (DSH) has often been used as a broader term for self-injurious behavior that includes both direct and indirect damage to an individual’s body, independently of suicidal intention [2]. Non-suicidal self-injury (NSSI) is a subcategory of self-injurious behavior representing the direct, deliberate destruction of one’s own body tissue (e.g., cutting, burning, carving, and interfering with wound healing) in the absence of an intent to die [3, 4]. The differentiation between suicidal and non-suicidal self-harm, however, is not only a matter of self-reported intention [4]; the behaviors most commonly associated with suicide (e.g., self-poisoning, shooting, hanging) also differ from the behaviors typically associated with NSSI (e.g., cutting, burning, carving, banging). This means that the classification of self-injurious behavior into suicidal and non-suicidal may rely both on self-reported intention and observed behavior. Hereafter, we use the term NSSI when the self-reported intention and/or the nature of the behavior indicates that it is non-suicidal; otherwise, we use the broader terms self-injurious behavior or self-harm.

The prevalence of NSSI varies widely between different studies. In a systematic review of the prevalence of NSSI in non-clinical samples, Swannell et al. [5] reported that the lifetime prevalence is around 17% among adolescents, 13% among young adults, and 5.5% among adults. A comparison of 12 European countries reported a mean prevalence rate of 27.6% in adolescents, ranging from 17.1% in Hungary to 38.7% in France [6]. In Sweden’s currently largest (*N* = 3054) study on NSSI in youth (15–17 years; [7]), 35.6% of participants reported at least one episode of NSSI during the preceding year. Of these, 6.7% met the Diagnostic and Statistical Manual of Mental Disorders, 5th edition (DSM-5) criteria for a suspected NSSI disorder; this prevalence was significantly higher among girls (11.1%) than among boys (2.3%).

Despite the high variation in the prevalence of NSSI across previous studies, the results clearly indicate that it is a public health concern [8]. Although NSSI may peak in mid-adolescence [9] and diminish afterward [for reviews see 10, 11], for a substantial number of young people, especially women, it may be long-lasting [12]. Furthermore, there is extensive evidence from cross-sectional and longitudinal studies that NSSI during adolescence has a strong association with concurrent and subsequent suicide ideation/attempts [e.g., 13, 14, 15], psychological symptoms (e.g., depression, anxiety, borderline personality disorder [BPD]; 16, 17]), and other psychosocial dysfunctions (e.g., cognitive vulnerability, sequelae from child sexual abuse, interpersonal distress; [e.g., 18, 19, 20]).

Despite a surge in research on NSSI over the last decade, there is still a relative scarcity of longitudinal studies of the course of NSSI from adolescence to young adulthood. To our knowledge, three longitudinal studies have examined the course of self-injurious behavior from adolescence to young adulthood in a population-based cohort: one from Norway [21], one from Australia [12], and one from the UK [22]. Although all three studies found that self-injurious behavior declined from adolescence to young adulthood and that self-injurious behavior in adolescence had negative psychological correlates in young adulthood, they all relied on a single item to measure self-harm, which is an important caveat limiting the generalizability of the results. For example, the very low prevalence of self-harm among both adolescents (2.4%; *M*_age_ = 16.5) and young adults (2.2%; *M*_age_ = 22.1) found by Wichstrom [21] is probably due to the use of a single item to measure self-harm—an item that focused on taking overdoses, rather than NSSI. Wichstrom [21] further found that youths’ (*M*_age_ = 16.5 years) self-harm, sexual history and sexual interest, and social support were significant predictors of self-harm in young adulthood (*M*_age_ = 22.1 years).

In the Australian study, Moran et al. [12] also relied on a single question asking about self-injurious behavior, although this question did not mention any specific forms of self-harm. They found a relatively low prevalence of self-injurious behavior among adolescents (about 8%), with about 80% of those who had reported self-harm in adolescence reporting no further self-harm in young adulthood (i.e., only 3% reported self-harm in adulthood). Their longitudinal results showed that self-injurious behavior at age 14–15 predicted increased risk of substance abuse and dependence at age 28–29 [23]. Borschmann et al. [24], utilizing the same sample, reported an increased risk of common mental problems (e.g., depression, anxiety), substance dependence, financial hardship, a history of divorce or separation, and multiple social disadvantages at age 35 years. Again, however, the low base rates are probably due to the reliance on a single question to measure self-injurious behavior, which represents a severe limitation of these studies.

The third study [22] similarly relied on a single item to measure self-injurious behavior in the UK. However, two specific forms of self-harm were explicitly mentioned in this question: taking overdoses and self-cutting. Their results showed that, although 19% of the sample reported a lifetime history of self-harm at age 16 years, this decreased to about 7% by age 21. They also found that participants who self-harmed with and without suicidal intent at age 16 years showed an increased risk of developing mental health problems, future self-harm, and problematic substance use by early adulthood (at age 18–21).

The present study focuses specifically on NSSI, rather than on self-injurious behavior in a wider sense. Research on NSSI may use either single-item measures or questionnaire that ask about several specific forms of NSSI, such as cutting, carving, burning, biting, and punching oneself. Questionnaires of the latter kind produce considerably higher prevalence rates [6]. This was shown most clearly by Zetterqvist et al. [7], who administered both a single-item question and a questionnaire asking for several different forms of NSSI at the same occasion to a large randomized community sample of adolescents. For the single-item question (“Have you ever…”), the prevalence rate of NSSI was 17.2%, but for the more detailed questionnaire, the prevalence rate more than doubled to 35.6%.

As argued by Lundh et al. [25], measuring self-injurious behavior with a single question relies heavily on the sensitivity of that question. They also questioned whether a single question is a sufficiently strong cue for the retrieval of all relevant varieties of self-harm from participants’ memories, and suggested that if one or a few forms of self-harm (e.g., taking overdoses, self-cutting) are specifically mentioned, this will affect what kind of memory retrieval processes are activated in respondents. Accordingly, one-item measures may be under-inclusive (i.e., produce too many false negatives) and thereby run the risk of failing to detect health-relevant forms of self-harm. On the other hand, questionnaires that ask for many different forms of self-harm might run the risk of being over-inclusive (i.e., produce too many false positives) or of producing affirmative responses that are irrelevant to future health outcomes. Increased clarity on this topic might be practically important for health-related policies.

Another limitation of the above-mentioned longitudinal studies is that they failed to make a distinction between occasional and repetitive self-injurious behaviors. It has been suggested that only repetitive self-harm over a lengthy time in adolescence is associated with negative longitudinal outcomes for psychological health [9]. As argued by Hawton et al. [26], single or occasional episodes of self-harm in adolescence could merely be a temporary behavioral testing of limits or the result of imitating peers. However, even occasional engagement in self-harm has clearly been linked to emotional and behavioral problems in adolescence [27]. Whether there are any longitudinal effects of occasional NSSI in adolescence—a question also highlighted by Whitlock and Selekman [28]—remains to be studied. Furthermore, these studies covered only the negative aspects of well-being and psychological functioning. As posited by the dual continuum model [29], these negative aspects are related to, but essentially separated from, the positive aspects of mental health such as satisfaction with life and flourishing. Whether and how different frequencies of NSSI in adolescence influence the positive and negative aspects of mental health later in life should also be studied.

The aims of the present study were to (1) estimate the overall prevalence of any NSSI and repetitive NSSI in adolescence and young adulthood and (2) study whether adolescents’ engagement in NSSI with varying frequency is associated with long-term imprints on individuals’ mental health. In pursuit of the latter aim, we examined the relationships between NSSI in early adolescence and both positive (i.e., satisfaction with life, flourishing) and negative (i.e., NSSI, depression, anxiety, stress, emotion dysregulation, psychiatric diagnoses) aspects of mental health 10 years later. Our use of a nine-item questionnaire to measure different forms of NSSI also made it possible to study whether not only repetitive, but also infrequent or occasional NSSI is associated with negative outcomes in young adulthood. In accordance with previous literature on NSSI (e.g., [30, 31]), we defined repetitive NSSI as at least five instances; this definition is also partly in line with a criterion for diagnosing NSSI in the DSM-5 [32]—namely, engagement in NSSI for 5 or more days in the past year. Although this choice of cutoff is by necessity arbitrary, using a similar cutoff to that used in previous research makes it easier to compare findings between different studies.

Using a 10-year, three-wave [adolescent phase: T1 (*M*_age_ = 13.7) and T2 (*M*_age_ = 14.8); adult phase: T3 (*M*_age_ = 25.3)] prospective follow-up study of a large community sample of Swedish youth, we estimated the overall prevalence of any NSSI and repetitive NSSI at three time points. We then explored the associations of different longitudinal frequency patterns of NSSI during adolescence with positive and negative indicators of mental health in young adulthood. Here, we differentiated between infrequent NSSI (1–4 episodes at T1 and/or T2, and no more than 4 episodes at either T1 or T2), unstable repetitive NSSI (5 or more episodes at either T1 or T2), and stable repetitive NSSI (5 or more episodes at both T1 and T2). Because our main research question was whether adolescents’ engagement in NSSI (of varying frequency and persistence) has a long-term imprint on individuals’ mental health later in life, we controlled for the confounding effects of mental health in adolescence (as measured by the Strength and Difficulties Questionnaire [33], one of the most widely used mental health screening tools among youth). We also controlled for gender, because of the commonly reported gender differences in self-reported psychopathology and because gender was a strong predictor of NSSI in a recent review by Valencia-Agudo et al. [34].

Based on previous longitudinal studies [12, 22], we expected that (1) the prevalence of NSSI would decrease from adolescence to young adulthood, and (2) that individuals who reported stable repetitive NSSI in adolescence would report significantly lower mental health in young adulthood than individuals who reported no NSSI in adolescence. Additionally, one of our main research questions was whether even infrequent NSSI, or unstable repetitive NSSI, would be associated with negative health outcomes after 10 years.

## Methods

### Participants

The data collection took place at three time points, starting in 2007 in a municipality in southern Sweden with about 40,000 inhabitants. In 2007 (T1), we addressed all regular school students in Grades 7 and 8 in this municipality; at T2, 1 year later, we addressed all regular school students in Grades 8 and 9. At T1, 93% of all students [mean (SD) age 13.7 (0.68); 50.1% girls; 15.2% with foreign background^1^] participated, and at T2, 90% participated [mean (SD) age 14.8 (0.69); 51.1% girls, 14.8% with foreign background]. In total, 909 (86%) of the students participated at both time points. In 2017, at T3, a 10-year follow-up was carried out, addressing all individuals who were eligible for the two earlier data collection points (*N* = 1109). Five hundred and fifty-seven individuals participated [response rate: 50.2%; mean (SD) age 25.3 (0.68); 59.2% women]. Of the 909 individuals with data from both T1 and T2, 475 (52.3%) participated in the third data collection, and these constitute the longitudinal sample studied in the current study. As seen in Table 1, most participants at T3 were married/cohabiting or were in a relationship (63%), had no children (89.4%), and were part- or full-time employees (60.5%).

**Table 1:** Descriptive statistics for the participants at T3

Variable	Total	Women	Men
Age (SD)	25.3 (0.68)	25.3 (0.52)	25.4 (0.68)
Marital status			
Single	203 (36.4%)	104 (31.8%)	99 (43.0%)
Married/cohabitant	280 (50.3%)	180 (55.0%)	100 (43.5%)
In a relationship	67 (12.0%)	39 (11.9%)	28 (12.2%)
Other	7 (1.3%)	4 (1.2%)	3 (1.3%)
Child status			
Yes	58 (10.4%)	46 (14.1%)	12 (5.2%)
No	498 (89.6%)	280 (85.9%)	218 (94.8%)
Number of children			
One	40 (69.0%)	31 (67.4%)	9 (75.0%)
Two or more	18 (31.0%)	15 (32.6%)	3 (25.0%)
Educational level			
Lower secondary education	18 (3.2%)	10 (3.1%)	8 (3.5%)
Upper secondary school	265 (47.7%)	145 (44.5%)	120 (52.2%)
Single university courses	48 (8.6%)	24 (7.4%)	24 (10.4%)
University degree (< 3 years)	38 (6.8%)	25 (7.7%)	13 (5.7%)
University degree (≥ 3 years)	172 (30.9%)	115 (35.3%)	57 (24.8%)
Other	15 (2.7%)	7 (2.1%)	8 (3.5%)
Current employment status			
Student	145 (26.2%)	85 (26.2%)	60 (26.3%)
Full-/part-time employment	334 (60.4%)	190 (58.5%)	144 (63.2%)
Unemployed	26 (4.7%)	13 (4.0%)	13 (5.7%)
On sick leave	11 (2.0%)	9 (2.8%)	2 (0.9%)
On parental leave	19 (3.4%)	19 (5.8%)	0 (0%)
Other	18 (3.3%)	9 (2.8%)	9 (3.9%)
Have you been on sick leave longer than 2 months?			
Yes	49 (9%)	37 (11.6%)	12 (5.3%)
No	497 (91%)	281 (88.4%)	216 (94.7%)
Have you been diagnosed with one or more psychiatric disorders?			
Yes	82 (14.8%)	67 (20.5%)	15 (6.6%)
No	473 (85.2%)	260 (79.5%)	213 (93.4%)
Above the cutoff on the McLean Screening Instrument for BPD^a^			
Yes	62 (11.4%)	48 (15.0%)	14 (6.2%)
No	484 (88.6%)	273 (85.0%)	211 (93.8%)

Of the 557 participants at T3, two reported different genders across the three time points. Their genders were set as those they reported at T3

^a^Seven or more “yes” responses on the McLean Screening Instrument for borderline personality disorder [45]

### Measures

#### Variables measured at T1, T2, and T3

*Self-injurious behavior* was measured at all three time points using the revised nine-item version of the Deliberate Self-Harm Inventory (DSHI-9r). This is a modified version of Gratz’s [35] Deliberate Self-Harm Inventory, which was developed in three steps. First, a short 16-item version was tested [25]; this was then shortened into a 9-item version with a new response format which was tested in a pilot study [36], which finally led to the development of the present version [37]. The respondents are asked to rate, on a 7-point scale ranging from 0 (never) to 6 (more than five times), how often they have engaged in nine forms of NSSI: (1) cutting wrists, arms or other body areas; (2) minor cutting (e.g., arms), causing bleeding; (3) punching/banging one’s head; (4) carving words, pictures, etc. into skin; (5) severe scratching, causing bleeding; (6) burning with cigarette, lighter or match; (7) sticking sharp objects into skin; (8) biting oneself, so that skin is broken; and (9) preventing wounds from healing) during the past 6 (T1/T2) or 12 (T3) months. The total score (range 0–54) is calculated by summing the item scores. Cronbach’s alpha values were 0.90 (T1), 0.90 (T2), and 0.81 (T3). At T3, we also added a separate question asking whether participants had attempted suicide in connection with harming themselves.

#### Variables measured at only T1 and T2

*Psychological difficulties* was measured using the total difficulties score on the Strengths and Difficulties Questionnaire–self-report version (SDQ-s; [33]), which is a summary score of 20 items asking about emotional symptoms, hyperactivity–inattention, conduct problems, and peer problems. All SDQ-s items are rated on a 3-point scale ranging from 0 (not true) to 2 (certainly true) and cover behavior occurring in the past 6 months. The Swedish version of the SDQ-s was empirically validated by Lundh et al. [38]. Cronbach’s alphas for the SDQ-s total difficulties score were 0.76 (T1) and 0.75 (T2).

#### Variables measured at T3

At T3, a number of instruments were used to measure positive and negative aspects of mental health, emotion regulation, sick leave, psychiatric diagnoses, and borderline personality features.

*Life satisfaction* was measured by the Satisfaction with Life Scale (SWLS; [39]), which consists of five items. Participants indicated how much they agreed or disagreed with each item using a 7-point scale ranging from 1 (strongly disagree) to 7 (strongly agree). Cronbach’s alpha for the scale was 0.92 in the present study.

*Flourishing *was measured by the Flourishing Scale (FS; [40]). The FS is an eight-item summary measure of an individual’s self-perceived success in important areas such as relationships, self-esteem, purpose, and optimism. Participants indicated how much they agreed or disagreed with each item using a 7-point scale ranging from 1 (strongly disagree) to 7 (strongly agree). The range of possible scores is from 8 to 56. Higher scores suggest that the person has more psychological resources and strengths. Cronbach’s alpha for the scale was 0.88 in the present study.

*Depression, anxiety, and stress* were measured using the Depression, Anxiety and Stress Scale (DASS-21; [41]) based on Lovibond and Lovibond [42], which contains 21 items (i.e., 7 items per construct). The items assessing depression (*α* = 0.90), anxiety (*α* = 0.79), and stress (*α* = 0.87) in the past month all used a 4-point scale ranging from 0 (never) to 3 (almost always).

*Emotion regulation* was measured using the Brief Difficulties in Emotion Regulation Scale (DERS-16), which was shortened from the original Difficulties in Emotion Regulation Scale [43] by Bjureberg et al. [44]. The DERS-16 taps several conceptual aspects of emotion dysregulation, including lack of emotional clarity, difficulties engaging in goal-directed behavior and controlling impulses, ineffective emotion regulation strategies, and non-acceptance of emotional responses. Participants indicated how often each of the 16 statements applied to them using a 5-point Likert scale ranging from 1 (almost never/0–10% of time) to 5 (almost always/91–100% of time). Cronbach’s alpha for the scale was 0.95.

*Sick leave for longer than 2 months* was assessed using a single item, to which participants answered either 1 (yes) or 0 (no). Being diagnosed with one or more psychiatric disorders was assessed by the question “Have you been diagnosed with one or more psychiatric disorders?” Participants answered with either 1 (yes) or 0 (no). Participants who responded affirmatively to this question were asked to describe the type of disorder(s).

*Borderline personality disorder* We used the McLean Screening Instrument for Borderline Personality Disorder (MSI-BPD; [45]) to screen for whether participants met the DSM-5 criteria for borderline personality disorder at T3. This self-report instrument contains ten items covering the nine diagnostic criteria for BPD in adulthood in the DSM-5. Participants responded to each item with either 1 (yes) or 0 (no). The MSI-BPD has sensitivity and specificity values above 0.90 for a cut- off score of ≥ 7 among people of age 25 years or younger [45]. Because the current study already included an extensive measure of NSSI and a separate question on suicide attempts, item 2, which targets deliberate self-harm and suicide attempts, was removed. To replace this item, we added one point to participants’ MSI-BPD score if they responded affirmatively to any of the corresponding questions on the DSHI-9r or to the question about suicide attempts. Cronbach’s alpha for the modified MSI-BPD was 0.80.

### Procedure and ethics

The data collection at T1 and T2 was conducted in collaboration with the municipal body of the study area and each of the regular schools therein. The headmaster of each school was contacted and gave consent to their school’s participation. Information describing the main objectives of the study was sent to the parents of all students, as well as handed out directly to students in school. All data were collected at school as part of a separate lecture hour. Teachers were present for the lecture hour, but did not take part in questionnaire administration, which was conducted by research assistants from Lund University. Adolescents were told that they were free to refrain from participation, and that they should not write their names anywhere on the questionnaire to ensure their confidentiality. Numeric codes were used to identify participants and to match the data from T1 and T2.

To conduct the follow-up at T3, we used the participants’ names from the original class lists of 2007–2008 (in accordance with approval from the Regional Ethics Committee; see below) to get their official person numbers from the school registers. We then contacted the Swedish state’s personal address register (SPAR) to identify their present locations. After receiving the current personal addresses of the participants, letters describing the purpose and procedure of the follow-up were sent to all participants. The participants completed a questionnaire either in the form of a confidential web survey designed using the Lund University survey system, Survey & Report, or a paper-and-pencil questionnaire sent by post. Numeric codes were used throughout the study on all study documents to identify participants (thereby preserving confidentiality). After completing the questionnaire, participants received two cinema tickets or four lottery tickets as compensation.

Ethical approval was obtained from the Regional Ethics Committee at Lund University in 2006 (Dnr 2006/49, for data collections at T1 and T2) and in 2016 (Dnr 2016/1059, for data collection at T3). For data collection at T1 and T2, informed consent was obtained by sending written information to the parents of all students and by handing out information directly to all students at school. The information sheets described the study and stated that participation was entirely voluntary. Students could refrain from participating by telling their teachers, or parents could contact the teachers or researchers directly to announce that their children should not participate. We considered this passive consent procedure as the most appropriate means of collecting informed consent under the circumstances [46]. For data collection at T3, potential participants were also informed that their participation was voluntary.

### Statistical analysis

Our analyses were conducted on a complete cases dataset. To ensure result trustworthiness, first, attrition analyses were performed by comparing the responders and non-responders at T3 in terms of all variables measured at T1 and T2. For the attrition analysis, between-group (i.e., responders vs. non-responders) comparisons were conducted using the independent-samples *t* test and Chi-square test for continuous and categorical variables, respectively. Further, we conducted multiple imputation (MI) by chained equations using the mice 3.7.0 command [47] in R version 3.6.2 to create multiple copies of datasets, wherein the missing values were sampled from their predictive distribution. Overall, we generated 50 imputations for each outcome of interest.

Regression analyses were used to examine the associations between different frequency patterns of NSSI in adolescence and mental health indicators in young adulthood. For all types of regression analysis, the original categorical NSSI pattern variable, which had four different values, was recoded into three dichotomous dummy variables with the No NSSI group as the reference, and entered in the analyses at Step 1. Participants’ gender [1 = girl/woman, 0 = boy/man] and psychological difficulties measured in adolescence were entered at Step 2 to see whether the associations between different frequency patterns of NSSI in adolescence and mental health indicators in young adulthood diminish or disappear. Logistic regression analysis was performed for dichotomous dependent variables [i.e., being on sick leave longer than 2 months (1 = yes, 0 = no), being diagnosed with one or more psychiatric disorders (1 = yes, 0 = no), and scoring above the cutoff (i.e., ≥ 7) of the MSI-BPD (1 = yes, 0 = no)], while multiple linear regression analysis was performed for the continuous dependent variables (i.e., positive and negative indicators of mental health and NSSI). Finally, multinomial regression was used to examine the associations between different frequency patterns of NSSI in adolescence and young adulthood. All the above-mentioned analyses were conducted in SPSS Statistics 25 (IBM Corp., Armonk, NY).

## Results

### Attrition analyses

Attrition analyses were performed by comparing the responders and non-responders at T3 in terms of all variables measured at T1 and T2. More specifically, for the 1070 individuals who had data on T1 and/or T2, we compared those who responded at T3 (*N* = 541) with those who did not respond at T3 (*N* = 529) (for details, see the attrition report of the project, [48]). Of the variables used in the present study, significantly more women responded to the survey at T3 [T1 and T2: 51%, T3: 58.4%; *χ*^2^(1) = 29.30, *p* < 0.001]. No significant differences were found for either self-injurious behavior or psychological difficulties. At T1, the means (SDs) for NSSI for the responders and non-responders were, respectively, 3.63 (8.92) and 3.04 (6.30), *t*(910.1) = − 1.19, *p* = 0.235. At T2, the corresponding means (SDs) were 3.77 (8.50) and 3.33 (7.98), *t*(944) = − 0.82, *p* = 0.412. At T1, the means (SDs) for psychological difficulties for the responders and non-responders, respectively, were as follows: 9.90 (5.36) and 10.30 (4.65), *t*(930) = − 1.22, *p* = 0.223. At T2, these means (SDs) were 10.43 (5.23) and 10.59 (4.77), *t*(922) = − 0.50, *p* = 0.617.

### Prevalence of NSSI in adolescence (T1 and T2) and young adulthood (T3)

Table 2 shows the prevalence rates of any form of NSSI (≥ 1 instance) and repetitive NSSI (≥ 5 instances) by time point and gender. The McNemar tests revealed that the overall prevalence rates declined significantly between adolescence and young adulthood (*p* < 0.001 for both T1 vs. T3 and T2 vs. T3). While approximately 40% of adolescents reported at least one instance of NSSI at T1 and T2, the percentage at T3 was markedly lower (18.7%). Repetitive NSSI also decreased – from approximately 18% at T1 and T2 to 10% at T3. While significant gender differences were found for reporting at least one instance of NSSI at T1 (*χ*^2^ = 4.36, *p* = 0.037), T2 (*χ*^2^ = 14.99, *p* < 0.001), and T3 (*χ*^2^ = 5.83, *p* = 0.015), the gender difference in repetitive NSSI was significant at T2 (*χ*^2^ = 15.46, *p* < 0.001), but not at T1 or T3. Three out of the 93 (3.2%) participants who reported NSSI also reported suicide attempts during the preceding year at T3.

**Table 2:** Prevalence (%) and mean (SD) of non-suicidal self-harm in girls/women and boys/men at the three time points

	Girls/women	Boys/men	Total
Any form of NSSI, *n* (%)			
T1	221/493 (44.8%)	187/489 (38.2%)	408/982 (41.5%)
T2	241/495 (48.7%)	176/480 (36.7%)	419/979 (42.8%)
T3	72/327 (22.0%)	32/229 (14.0%)	104/556 (18.7%)
Repetitive NSSI^a^, *n* (%)			
T1	102/493 (20.7%)	79/489 (16.2%)	181/982 (18.4%)
T2	127/495 (25.7%)	76/480 (15.8%)	205/979 (20.9%)
T3	37/327 (11.3%)	21/229 (9.2%)	58/556 (10.4%)
NSSI, total score, M (SD)			
T1	4.24 (9.19)	2.60 (6.42)	3.47 (8.07)
T2	4.54 (9.05)	2.85 (8.19)	3.78 (8.85)
T3	1.69 (5.09)	1.10 (4.38)	1.45 (4.82)

*NSSI* non-suicidal self-injury

^a^Repetitive NSSI = at least five instances of non-suicidal self-injury. Across the three time points, participants with no more than three missing values on the non-suicidal self-harm measure were included for data analysis; missing values were interpreted conservatively as the absence of self-harming behavior (i.e., imputing 0). As such, there were data available on NSSI for 983 participants at T1, 979 at T2, and 556 at T3

### NSSI frequency patterns in adolescence

**Fig. 1:**
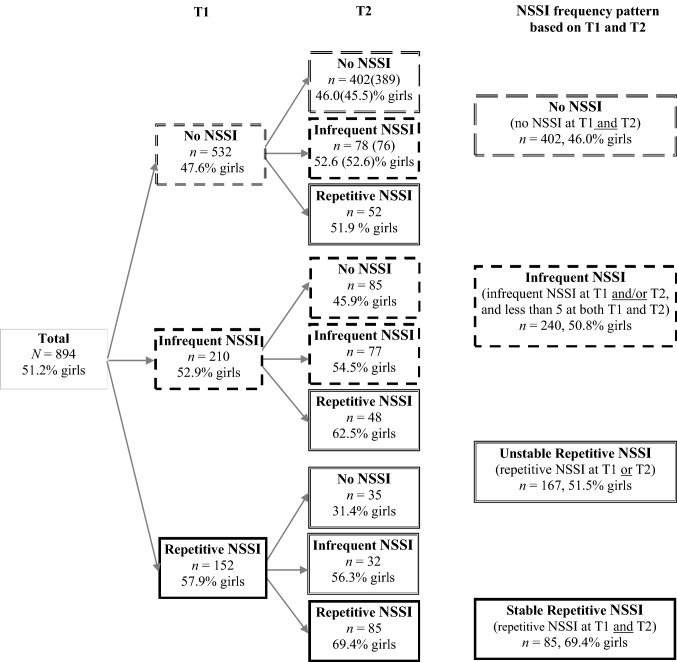
NSSI frequencies and frequency patterns in adolescence. *NSSI* = non-suicidal self-injury.* Infrequent NSSI* = 1–4 episodes of self-harm. Repetitive NSSI ≥ 5 episodes

Of the 909 individuals who responded at both T1 and T2, there were full data on NSSI for 894. Based on their NSSI frequency at T1 and T2, we assigned these participants to one of four groups: the no NSSI group (no reported NSSI at T1 and T2), the infrequent NSSI group (at least 1 episode of NSSI at T1 and/or T2, and no more than 4 episodes at either T1 or T2), the unstable repetitive NSSI group [repetitive (≥ 5 episodes) NSSI at either T1 or T2], and the stable repetitive NSSI group (repetitive NSSI at both T1 and T2). As shown in Fig. 1, although 402 (45%) adolescents did not report any NSSI during adolescence and 240 (26.8%) reported infrequent NSSI, a significant proportion of the adolescents reported repetitive NSSI at one of the two time points (*n* = 167, 18.7%) or at both time points (*n* = 85, 9.5%). Importantly, while the first three NSSI frequency groups had a rather equal gender distribution, the stable repetitive NSSI pattern was most frequent among women (69.4%).

### Are frequency patterns of NSSI in adolescence related to psychological health in adulthood?

Regression analyses were used to examine the associations between different frequency patterns of NSSI in adolescence and various mental health indicators in young adulthood while controlling for gender and psychological difficulties in adolescence. We recoded the four NSSI frequency patterns into three dichotomous dummy variables with the no NSSI pattern as the reference. At Step 1, these NSSI frequency patterns were included as predictors in the regression models. At Step 2, in addition to the NSSI frequency patterns, the control variables were also included. Different mental health indicators in young adulthood were used as outcome variables (see Tables 3 and 5 for the descriptive statistics).

**Table 3:** Dichotomous mental health outcomes in young adulthood for participants with different NSSI frequency patterns in adolescence

NSSI frequency pattern in adolescence	Mental health indicator in young adulthood		
	On sick leave for longer than 2 months	Diagnosed with psychiatric disorder(s)	Above cutoff on McLean screening instrument for BPD
No NSSI, total	11/224 (4.9%)	21/224 (9.4%)	11/220 (5.0%)
Girls	6/116 (5.2%)	19/117 (16.2%)	10/115 (8.7%)
Boys	5/108 (4.6%)	2/107 (1.9%)	1/105 (1%)
Infrequent NSSI, total	8/124 (6.5%)	15/125 (12.0%)	15/124 (12.1%)
Girls	5/68 (7.4%)	10/68 (14.7%)	9/67 (13.4%)
Boys	3/56 (5.4%)	5/57 (8.8%)	6/57 (10.5%)
Unstable repetitive NSSI, total	7/69 (10.1%)	12/72 (16.7%)	8/71 (11.3%)
Girls	5/42 (11.9%)	9/45 (20.0%)	5/44 (11.4%)
Boys	2/27 (7.4%)	3/27 (11.1%)	3/27 (11.1%)
Stable repetitive NSSI, total	11/52 (21.2%)	15/53 (28.3%)	12/50 (24.0%)
Girls	11/42 (26.2%)	15/43 (34.9%)	11/41 (26.8%)
Boys	0/10 (0%)	0/10 (0%)	1/9 (11.1%)

Values are numerators/denominators (%)

*NSSI* = non-suicidal self-injury, *BPD* = borderline personality disorder

The results of the regression analyses, summarized in Table 4, indicated that compared to those who did not report any NSSI in adolescence, adolescents with stable repetitive NSSI had a significantly increased odds of being on sick leave for longer than 2 months [odds ratio (OR) 5.20, 95% confidence interval (CI) (2.11, 12.78)], being diagnosed with one or more psychiatric disorders [OR 3.82, 95% CI (1.81, 8.06)], and scoring above the cutoff on the MSI-BPD [OR 6.00, 95% CI (2.47, 14.58)]. When the relationships were controlled for gender and psychological difficulties in adolescence, adolescents with stable repetitive NSSI still had significantly increased odds of scoring above the cutoff on the MSI-BPD [OR 2.99, 95% CI (1.04, 8.60)] 10 years later.

**Table 4:** Results of logistic regression analysis predicting mental health in young adulthood

NSSI frequency pattern in adolescence as predictor^a^	*b* (SE)	Wald	*p*	OR	95% CI	
					Lower	Upper
On sick-leave longer than 2 months (yes = 1, no = 0)						
*Step 1*						
Infrequent NSSI	0.29 (0.48)	0.37	0.546	1.34	0.52	3.41
Unstable repetitive NSSI	0.78 (0.51)	2.40	0.121	2.19	0.81	5.88
Stable repetitive NSSI	1.65 (0.46)	12.87	< 0.001	5.20	2.11	12.78
			*R*^2^ = 0.064 (Nagelkerke)			
*Step 2*						
Infrequent NSSI	0.14 (0.49)	0.08	0.775	1.15	0.44	3.00
Unstable repetitive NSSI	0.51 (0.53)	0.93	0.335	1.67	0.59	4.71
Stable repetitive NSSI	1.05 (0.56)	3.57	0.059	2.87	0.96	8.54
SDQ total	0.06 (0.04)	2.31	0.128	1.06	0.98	1.15
Gender	0.52 (0.40)	1.75	0.186	1.69	0.78	3.66
		*R*^2^ = 0.085 (Nagelkerke)				
Diagnosed with one or more psychiatric disorders (yes = 1, no = 0)						
*Step 1*						
Infrequent NSSI	0.28 (0.36)	0.60	0.441	1.32	0.65	2.66
Unstable repetitive NSSI	0.66 (0.39)	2.85	0.091	1.93	0.90	4.16
Stable repetitive NSSI	1.34 (0.38)	12.32	< 0.001	3.82	1.81	8.06
		*R*^2^ = 0.047 (Nagelkerke)				
*Step 2*						
Infrequent NSSI	0.09 (0.37)	0.06	0.803	1.10	0.53	2.29
Unstable repetitive NSSI	0.26 (0.42)	0.39	0.534	1.30	0.57	2.98
Stable repetitive NSSI	0.50 (0.46)	1.15	0.284	1.64	0.66	4.06
SDQ total	0.07 (0.03)	5.23	0.022	1.08	1.01	1.15
Gender	1.38 (0.37)	14.32	< 0.001	3.97	1.95	8.12
		*R*^2^ = 0.135 (Nagelkerke)				
Above cutoff on the McLean screening instrument for BPD (7 or more yes answers = 1, 0–6 yes answers = 0)						
*Step 1*						
Infrequent NSSI	0.96 (0.41)	5.39	0.020	2.62	1.16	5.89
Unstable repetitive NSSI	0.88 (0.49)	3.28	0.070	2.41	0.93	6.26
Stable repetitive NSSI	1.79 (0.45)	15.63	< 0.001	6.00	2.47	14.58
				*R*^2^ = 0.072 (Nagelkerke)		
*Step 2*						
Infrequent NSSI	0.81(0.43)	3.66	0.056	2.25	0.98	5.18
Unstable repetitive NSSI	0.55 (0.51)	1.13	0.288	1.73	0.63	4.73
Stable repetitive NSSI	1.09 (0.54)	4.11	0.043	2.99	1.04	8.60
SDQ total	0.07 (0.04)	3.35	0.067	1.07	1.00	1.15
Gender	0.75 (0.37)	4.09	0.043	2.12	1.02	4.38
		*R*^2^ = 0.107 (Nagelkerke)				

*NSSI* = non-suicidal self-injury

^a^The four NSSI patterns were recoded into three mutually exclusive dichotomous dummy variables with “No NSSI at T1 and T2” as the reference. Gender is coded as 1 = girl, 0 = boy. The analyses at Step 2 were controlled for participants’ SDQ total (i.e., psychological difficulties) measured in adolescence, and gender

Next, we examined the associations between the NSSI frequency patterns and both positive (life satisfaction and flourishing) and negative indicators (depression, anxiety, stress, emotional dysregulation, and NSSI) of mental health in young adulthood (see Table 5 for the descriptive statistics). The results of the multiple regression analyses indicated that stable repetitive NSSI in adolescence was strongly and significantly associated with poorer mental health 10 years later (see Table 6). Compared to those who did not report any NSSI in adolescence, participants with stable repetitive NSSI reported significantly lower life satisfaction (*b* = − 2.63, *t* = − 2.36, *p* = 0.019) and flourishing (*b* = − 3.36, *t* = − 2.97, *p* = 0.003), and significantly higher levels of stress (*b* = 3.95, *t* = 5.47, *p* < 0.001), anxiety (*b* = 2.58, *t* = 4.72, *p* < 0.001), and depression (*b* = 2.46, *t* = 3.60, *p* = 0.001). The associations were particularly strong for emotional dysregulation (*b* = 13.54, *t* = 6.49, *p* < 0.001) and episodes of NSSI in young adulthood (*b* = 4.03, *t* = 5.99, *p* < 0.001). When controlling for gender and psychological difficulties, participants with stable repetitive NSSI still showed significantly higher levels of stress (*b* = 1.67, *t* = 2.08, *p* = 0.038), anxiety (*b* = 1.31, *t* = 2.10, *p* = 0.037), emotional dysregulation (*b* = 7.36, *t* = 3.14, *p* = 0.002), and self-injurious behavior in young adulthood (*b* = 3.42, *t* = 4.40, *p* < 0.001) compared to the non-NSSI individuals. With statistical correction for the number of significance tests, the following results were quite robust: Adolescents showing stable repetitive NSSI showed increased emotion dysregulation and increased NSSI as young adults, even when gender and psychological difficulties were controlled for.

**Table 5:** Means (SDs) of the continuous mental health outcomes in young adulthood for participants with different NSSI frequency patterns in adolescence

NSSI frequency pattern in adolescence	*n*	Mental health indicators in young adulthood						
		Life Satisfaction	Flourishing	Stress	Anxiety	Depression	NSSI, total score	Emotion dysregulation
No NSSI, total	225	24.63 (7.06)	47.43 (7.12)	5.37 (4.12)	2.29 (2.79)	2.80 (4.01)	0.72 (4.24)	30.12 (12.53)
Girls	117	25.27 (6.27)	47.54 (6.93)	6.51 (4.43)	2.59 (3.16)	3.12 (4.01)	0.75 (3.74)	32.37 (13.65)
Boys	108	23.93 (7.80)	47.32 (7.35)	4.13 (3.35)	1.97 (2.28)	2.46 (3.99)	0.69 (4.74)	27.68 (10.74)
Infrequent NSSI, total	125	23.80 (7.13)	46.03 (7.85)	6.79 (5.09)	3.57 (4.08)	4.43 (4.78)	1.04 (3.06)	33.60 (14.41)
Girls	68	25.06 (6.61)	46.86 (7.38)	7.07 (5.04)	3.42 (4.09)	4.30 (4.35)	1.37 (3.80)	34.18 (15.17)
Boys	57	22.30 (7.49)	45.04 (8.34)	6.44 (5.16)	3.74 (4.09)	4.58 (5.29)	0.65 (1.79)	32.91 (13.55)
Unstable repetitive NSSI, total	72	22.51 (7.74)	46.10 (6.83)	7.68 (5.17)	4.25 (4.25)	4.78 (4.83)	1.18 (3.66)	35.88 (14,567)
Girls	45	23.20 (6.92)	46.78 (5.78)	8.64 (5.06)	4.55 (4.00)	4.70 (4.29)	1.09 (2.72)	37.82 (13.19)
Boys	27	21.37 (8.96)	44.96 (8.30)	6.07 (5.03)	3.74 (4.66)	4.91 (5.71)	1.33 (4.91)	32.65 (16.60)
Stable repetitive NSSI, total	53	21.99 (8.18)	44.08 (8.21)	9.32 (5.65)	4.87 (4.27)	5.27 (5.14)	4.75 (7.60)	43.65 (15.07)
Girls	43	21.30 (8.28)	44.12 (8.00)	10.00 (5.78)	5.19 (4.37)	5.63 (5.15)	4.56 (7.72)	44.62 (16.11)
Boys	10	25.00 (7.39)	43.90 (9.53)	6.40 (4.12)	3.50 (3.66)	3.70 (5.08)	5.60 (7.40)	39.47 (8.78)

*NSSI* = non-suicidal self-injury

**Table 6:** Results of multiple regressions predicting mental health in young adulthood

Adolescence variables as predictors	Mental health indicators in young adulthood as outcome variables															
	Life satisfaction				Flourishing				Stress				Anxiety			
	*b* (CI)	SE	*β*	*p*	*b* (CI)	SE	*β*	*p*	*b* (CI)	SE	*β*	*p*	*b* (CI)	SE	*β*	*p*
*Step 1*																
Infrequent NSSI	− 0.83 (− 2.43, 0.78)	0.82	− 0.05	0.312	− 1.40 (− 3.03, 0.22)	0.83	− 0.08	0.090	1.42 (0.38, 2.46)	0.53	0.13	0.007	1.28 (0.49, 2.06)	0.40	0.15	0.001
Unstable repetitive NSSI	− 2.11 (− 4.06, − 0.17)	0.99	− 0.10	0.034	− 1.36 (− 3.31, 0.64)	1.00	− 0.06	0.184	2.31 (1.05, 3.57)	0.64	0.17	< 0.001	1.96 (1.01, 2.91)	0.48	0.19	< 0.001
Stable repetitive NSSI	− 2.63 (− 4.83, − 0.44)	1.12	− 0.11	0.019	− 3.36 (− 5.58, − 1.13)	1.13	− 0.14	0.003	3.95 (2.53, 5.38)	0.72	0.25	< 0.001	2.58 (1.51, 3.65)	0.55	0.22	< 0.001
*R*^2^	0.02				0.02				0.07				0.07			
*Step 2*																
Infrequent NSSI	0.22 (− 1.36, 1.79)	0.80	0.01	0.788	− 0.41 (− 2.03, 1.20)	0.82	− 0.02	0.615	0.88 (− 0.25, 1.80)	0.52	0.08	0.090	0.94 (0.15, 1.73)	0.40	0.11	0.020
Unstable repetitive NSSI	− 0.40 (− 2.35, 1.56)	1.00	− 0.02	0.691	0.33 (− 1.67, 2.33)	1.02	0.02	0.747	1.26 (0.15, 2.72)	0.65	0.09	0.053	1.35 (0.36, 2.33)	0.50	0.13	0.008
Stable repetitive NSSI	0.70 (− 1.26, 3.71)	1.24	0.03	0.575	− 0.90 (− 2.58, 2.39)	1.26	− 0.00	0.943	1.67 (0.01, 3.20)	0.81	0.11	0.038	1.31 (0.08, 2.53)	0.62	0.11	0.037
SDQ total	− 0.49 (− 0.65, − 0.34)	0.08	− 0.32	< 0.001	− 0.47 (− 0.52, − 0.11)	0.08	− 0.30	< 0.001	0.22 (0.12, 0.38)	0.05	0.22	< 0.001	0.15 (0.07, 0.23)	0.04	0.20	< 0.001
Gender	1.61 (0.31, 2.98)	0.66	0.11	0.016	1.06 (− 0.27, 2.39)	0.68	0.07	0.116	1.94 (0.65, 2.48)	0.43	0.20	< 0.001	0.42 (− 0.24, 1.07)	0.33	0.06	0.212
*R*^2^	0.10				0.09				0.15				0.10			

Adolescence variables as predictors	Mental health indicators in young adulthood as outcome variables											
	Depression				DSH				Emotion dysregulation			
	*b* (CI)	SE	*β*	*p*	*b* (CI)	SE	*β*	*p*	*b* (CI)	SE	*β*	*p*
*Step 1*												
Infrequent NSSI	1.62 (0.64, 2.61)	0.50	0.16	0.001	0.32 (− 0.65, 1.28)	0.49	0.03	0.521	3.48 (0.49, 6.48)	1.53	0.11	0.023
Unstable repetitive NSSI	1.98 (0.79, 3.17)	0.61	0.16	0.001	0.46 (− 0.72, 1.63)	0.60	0.04	0.445	5.77 (2.13, 9.40)	1.85	0.15	0.002
Stable repetitive NSSI	2.46 (1.12, 3.81)	0.69	0.17	< 0.001	4.03 (2.71, 5.35)	0.67	0.28	< 0.001	13.54 (9.43, 17.64)	2.09	0.30	< 0.001
*R*^2^	0.05				0.07				0.09			
*Step 2*												
Infrequent NSSI	1.15 (0.16, 2.13)	0.50	0.11	0.023	0.15 (− 0.84, 1.14)	0.50	0.01	0.769	1.94 (− 1.04, 4.92)	1.52	0.06	0.202
Unstable repetitive NSSI	1.13 (− 0.10, 2.36)	0.63	0.09	0.072	0.16 (− 1.07, 1.39)	0.63	0.01	0.801	2.84 (− 0.87, 6.56)	1.89	0.07	0.133
Stable repetitive NSSI	0.73 (− 0.80, 2.26)	0.78	0.05	0.349	3.42 (1.89, 4.95)	0.78	0.24	< 0.001	7.36 (2.75, 11.98)	2.35	0.16	0.002
SDQ total	0.22 (0.12, 0.31)	0.05	0.22	< 0.001	0.08 (− 0.02, 0.17)	0.05	0.08	0.125	0.67 (0.37, 0.96)	0.15	0.22	< 0.001
Gender	0.29 (− 0.53, 1.11)	0.42	0.03	0.483	0.09 (− 0.73, 0.91)	0.42	0.01	0.829	3.62 (1.56, 6.09)	1.26	0.13	0.004
*R*^2^	0.09				0.08				0.14			

*NSSI* = non-suicidal self-injury. The four NSSI patterns were recoded into three mutually exclusive dichotomous dummy variables with “No NSSI at T1 and T2” as the reference. Gender is coded as 1 = girl, 0 = boy. The analyses at Step 2 were controlled for participants’ SDQ total (i.e., psychological difficulties) measured in adolescence and gender

Infrequent NSSI and unstable repetitive NSSI showed relatively weaker associations with mental health indicators in young adulthood, compared to stable repetitive NSSI. Still, individuals who had shown the former NSSI patterns during adolescence reported significantly higher stress (*b* = 1.42, *t* = 2.69, *p* = 0.007 and *b* = 2.31, *t* = 3.61, *p* < 0.001, for infrequent NSSI and unstable repetitive NSSI patterns, respectively), anxiety (*b* = 1.28, *t* = 3.20, *p* = 0.001 and *b* = 1.96, *t* = 4.05, *p* < 0.001), depression (*b* = 1.62, *t* = 3.24, *p* = 0.001 and *b* = 1.98, *t* = 3.26, *p* = 0.001), and emotion dysregulation (*b* = 3.48, *t* = 2.28, *p* = 0.023 and *b* = 5.77, *t* = 3.12, *p* = 0.002) in young adulthood, as compared with those who did not report any self-injurious behavior in adolescence. When controlling for gender and psychological difficulties in adolescence, the associations for anxiety (*b* = 0.94, *t* = 2.33, *p* = 0.020 and *b* = 1.35, *t* = 2.68, *p* = 0.008, for infrequent NSSI and unstable repetitive NSSI patterns, respectively) and depression (only for infrequent NSSI pattern, *b* = 1.15, *t* = 2.28, *p* = 0.023) remained significant. With statistical correction for the number of significance tests, the following finding was quite robust: Adolescents with unstable repetitive NSSI showed increased levels of anxiety as young adults, even when gender and psychological difficulties were controlled for.

Finally, multinomial regression was used to examine the associations between different NSSI frequency patterns in adolescence and those in young adulthood. As Table 7 shows, of the participants who reported infrequent NSSI or unstable repetitive NSSI in adolescence and had NSSI data in young adulthood, about 80% did not report any NSSI in young adulthood. Moreover, only 49% of those who reported stable repetitive NSSI in adolescence did not report any form of NSSI in young adulthood and about 36% reported repetitive NSSI both in adolescence and young adulthood. Of the three participants who reported suicide attempts within the past year, two reported repetitive NSSI at all three time points.

**Table 7:** Cross-tabulation of different NSSI frequency patterns in adolescence and young adulthood

NSSI frequency pattern in adolescence	NSSI frequency in adulthood		
	No NSSI at T3 (*N* = 389)	Infrequent NSSI at T3 (*N* = 39)	Repetitive NSSI at T3 (*N* = 47)
No NSSI (*n* = 225)	208/225 (92.4%)	6/225 (2.7%)	11/225 (4.9%)
Infrequent NSSI (*n* = 125)	98/125 (78.4%)	16/125 (12.8%)	11/125 (8.8%)
Unstable repetitive NSSI (*n* = 72)	57/72 (79.2%)	9/72 (12.5%)	6/72 (8.3%)
Stable repetitive NSSI (*n* = 53)	26/53 (49.1%)	8/53 (15.1%)	19/53 (35.8%)

*NSSI* = non-suicidal self-injury. Values are numerators/denominators (%)

The multinomial regression analyses (see Table 8) showed that adolescents with infrequent NSSI [OR 4.55, 95% CI (1.80, 11.51)], unstable repetitive NSSI [OR 4.95, 95% CI (1.76, 13.90)], and stable repetitive NSSI in adolescence [OR 9.14, 95% CI (3.06, 27.28)] had significantly greater odds of reporting infrequent NSSI in adulthood compared to adolescents who reported no NSSI in adolescence. Furthermore, compared to the no-NSSI pattern, the stable repetitive NSSI frequency pattern in adolescence had a particularly strong association with repetitive NSSI in young adulthood [OR 14.40, 95% CI (6.01, 34.51)]. As Table 8 shows, the results remained significant after controlling for gender and psychological difficulties in adolescence.

**Table 8:** Results of multinomial regression analysis predicting nssi frequency pattern in young adulthood

NSSI frequency and other variables in adolescence as predictors^a^	*b* (SE)	Wald	*p*	OR	95% CI	
					Lower	Upper
Infrequent NSSI vs. no NSSI at T3						
*Step 1*						
Infrequent NSSI	1.73 (0.49)	12.31	< 0.001	4.55	1.80	11.51
Unstable repetitive NSSI	1.60 (0.53)	9.23	0.002	4.95	1.76	13.90
Stable repetitive NSSI	2.21 (0.56)	15.74	< 0.001	9.14	3.06	27.28
*Step 2*						
Infrequent NSSI	1.38 (0.49)	8.16	0.004	3.99	1.54	10.32
Unstable repetitive NSSI	1.25 (0.56)	5.11	0.024	3.50	1.18	10.38
Stable repetitive NSSI	1.51 (0.65)	5.48	0.019	4.52	1.28	15.99
SDQ total	0.05 (0.05)	0.95	0.330	1.05	0.95	1.17
Gender	1.13 (0.45)	6.34	0.012	3.10	1.29	7.48
Repetitive NSSI vs. no NSSI at T3						
*Step 1*						
Infrequent NSSI	0.85 (0.45)	3.49	0.062	2.34	0.96	5.68
Unstable repetitive NSSI	0.84 (0.54)	2.42	0.120	2.31	0.80	6.64
Stable repetitive NSSI	2.67 (0.45)	35.78	< 0.001	14.40	6.01	34.51
*Step 2*						
Infrequent NSSI	0.79 (0.46)	2.95	0.086	2.21	0.90	5.44
Unstable repetitive NSSI	0.74 (0.56)	1.75	0.186	2.09	0.70	6.26
Stable repetitive NSSI	2.47 (0.54)	21.04	< 0.001	11.80	4.11	33.88
SDQ total	0.04 (0.05)	0.63	0.428	1.04	0.94	1.15
Gender	− 0.06 (0.38)	0.03	0.871	0.94	0.45	1.97

*NSSI* = non-suicidal self-injury

^a^The four NSSI patterns were recoded into three mutually exclusive dichotomous dummy variables with “No NSSI at T1 and T2” as the reference. Gender is coded as 1 = girl, 0 = boy. The analyses at Step 2 were controlled for participants’ SDQ total (i.e., psychological difficulties) measured in adolescence and gender

Supplementary Tables S1 and S2 present the results of the imputed data analysis. The findings were generally consistent with those from the complete case analysis.

## Discussion

This study examined the prevalence of self-injurious behavior at three time points from early adolescence to young adulthood, and studied the associations between various frequency patterns of NSSI in adolescence (infrequent, unstable repetitive, and stable repetitive) and mental well-being and functioning 10 years later. As part of the latter, we focused on both positive (i.e., satisfaction with life, flourishing) and negative (i.e., NSSI, depression, anxiety, stress, emotional dysregulation, being on sick leave, psychiatric diagnoses) aspects.

As expected, and in line with the results of other longitudinal studies [12, 22], we found that the prevalence of NSSI decreased from adolescence to young adulthood. However, the prevalence in young adulthood was still considerably higher compared to those reported by Moran et al. [12] and Mars et al. [49] for self-injurious behavior more generally. There are several possible explanations for these discrepancies. For example, Brunner et al. [27] mentioned that cultural differences could explain the differences in the prevalence of self-injurious behavior between different studies. In view of the cultural similarities between Sweden and Norway, however, it is unlikely that cultural differences are the main explanation for the large differences between Wichstrom’s [21] results in Norway and those in our study in Sweden. Another possibility, as mentioned in the introduction, is that the method of measuring self-injurious behavior plays a major role. Multiple-item measures of NSSI, such as the one used in the present study, tend to produce considerably higher prevalence rates than one-item measures, even when administered to the same sample at the same time point [7]. One possible reason for this phenomenon is that multiple-item measures produce more false positives than do single-item measures, and therefore may run the risk of producing affirmative responses that are irrelevant to future health outcomes. Another possible reason, however, is that multi-item measures are more sensitive, and may therefore be able to detect individuals at risk for mental ill-health that may be missed by single-item measures.

The results of this study generally support the latter interpretation. One of the main findings was that not only individuals who engaged in stable repetitive NSSI in early adolescence, but also those who engaged in infrequent or unstable repetitive NSSI during these early years reported significantly higher anxiety in young adulthood than did those without NSSI, even after controlling for gender and psychological difficulties in adolescence. Individuals who engaged in infrequent NSSI also demonstrated higher depression in young adulthood. The finding that adolescents with unstable repetitive NSSI showed increased levels of anxiety as young adults, even after controlling for gender and psychological difficulties, was particularly robust. Although the majority (about 80%) of individuals who showed infrequent or unstable repetitive NSSI patterns in adolescence did not report any self-harm in young adulthood, the results clearly showed that adolescents with these patterns had significantly increased odds of infrequent NSSI in young adulthood compared to those who never reported any NSSI. Moreover, although the results were non-significant, the CIs suggested that adolescents with infrequent NSSI (CI 0.98–5.18) had greater odds of scoring above the cutoff for BPD after 10 years. Altogether, these results suggest that NSSI in early adolescence might be an independent risk factor for negative health outcomes 10 years later.

Considering these results, it seems important to differentiate between what are *markers of a negative outcome* (a weaker interpretation) and what are *independent risk factors* (a stronger interpretation). The stronger interpretation requires that other risk factors (i.e., general psychological difficulties) be controlled for, whereas the weaker interpretation would not require this. Thus, according to the weaker interpretation, NSSI in early adolescence is a *marker*, or indicator, of negative health outcomes 10 years later. By contrast, according to the stronger interpretation, NSSI is an independent *risk factor* for negative health outcomes 10 years later. In this perspective, the present results suggest that even infrequent NSSI during adolescence is a marker for negative health outcomes ten years later, whereas it is more uncertain whether it is an independent risk factor (as these results were not equally statistically robust). However, the present results suggest that unstable repetitive self-harm is not only a marker for future negative outcome, but also an independent risk factor (at least for anxiety ten years later).

Thus, even infrequent NSSI in adolescence might be an early indicator of vulnerability to mental health problems in young adulthood. In other words, infrequent NSSI in adolescence is associated not only with short-term negative psychological outcomes including emotional and behavioral problems, as reported in Brunner et al. [27], but also with long-term negative outcomes. Multiple-item measures of NSSI thus seem sufficiently sensitive to detect individuals at risk for emotional problems in adulthood who might be missed by single-item measures. Studies using a single-item measure produce considerably lower prevalence rates, but might fail to detect adolescents at risk of developing mental health problems.

As expected, adolescents who engaged in stable repetitive NSSI in adolescence had a substantially increased risk of negative outcomes and lowered life satisfaction and flourishing in young adulthood. Even when psychological difficulties in adolescence were considered, these youths showed significantly increased risk of stress, anxiety, NSSI, and difficulties in emotion regulation after 10 years. Moreover, less than 50% of individuals who reported stable repetitive NSSI in adolescence did not report any NSSI in adulthood, while almost 36% of them reported repetitive NSSI both in adolescence and adulthood. These individuals also were significantly more likely to score above the cutoff for BPD after 10 years. Although only 1 individual out of the 15 with stable repetitive NSSI (in fact, the only one in the entire sample) reported being diagnosed with BPD, other participants with this NSSI pattern reported being diagnosed with other disorders, often multiple (e.g., ADHD, bipolar disorder, depression, post-traumatic stress disorder). Given that BPD affects 1–3% of the general population [50, 51] and is one of the most misdiagnosed mental health conditions [52], it is likely that other individuals in this study—especially among those with repetitive NSSI at all three time points and who scored above the cutoff on the MSI-BPD—suffered from BPD. At the same time, we should note that this cutoff was based on a screening instrument for BPD, and that the percentage of women who scored above this cutoff (15%) in the present study most certainly represents a considerable overestimation of the actual rate [50, 51].

### Limitations

First, only self-reported NSSI was assessed in this study; we did not check participants’ reports against hospital records or other sources. Although the lack of other data sources is a limitation, self-reports might still be the best way to obtain an accurate picture of self-injurious behavior, because only a relatively small portion of such behavior is revealed to clinical services [9].

A second possible limitation is that the DSHI-9r asks about the presence of self-harm only during the past 6 months (at T1 and T2) or 12 months (at T3), whereas the time intervals between T1 and T2, and between T2 and T3, were longer than these measurement intervals. This fact might have led to a failure to detect self-harm episodes that occurred when participants were 17–24 years old, before they were 13 years old, or in the 6 months directly after T1. If the purpose of the study had been to obtain detailed knowledge of the lifetime occurrence of self-harm episodes, our current methods would have been less appropriate. The present research, however, asked about how life is ten years afterwards for young adults who engaged in NSSI as adolescents. Although we might have failed to detect some adolescents who engaged in NSSI by phrasing the question in this way, we do not expect this to be a large group, given that more than 40% of adolescents actually reported having engaged in NSSI at T1 (and at T2). Altogether, therefore, in the context of our present research question, asking adolescents about self-harm during the six past months is not likely to present a major barrier to the interpretation of our results, even though we cannot be sure about this. The absence of data on NSSI between the ages of 17 and 25 years is a more serious limitation. For example, according to Gandhi et al. [53], there is a second peak wave of NSSI at about age 22 years; because of the lack of data from this time period, the present study cannot confirm this or contribute any further information. Greater attention should be paid to the risk factors in adolescence and young adulthood that could predict such late-onset NSSI.

Third, the sample had a large attrition ratio—slightly more than half the original sample responded to the 10-year follow-up. Although no clear systematic differences (other than gender) between responders and non-responders at the 10-year follow-up were found for any variable used in the present study, we did observe some differences in variables not considered in this study (e.g., direct aggression, body esteem), albeit with low effect sizes (Cohen’s *d* = 0.12–0.21). Similar response rates, however, were reported in the Norwegian longitudinal study by Sigurdson et al. [54] and in the ALSPAC study by Mars et al. [22], neither of which found any systematic differences between responders and non-responders.

Fourth, the absence of data on socioeconomic status (SES) in adolescence is a limitation. SES is an important determinant of health, and it would have been interesting to include SES in the analyses to add a sociological perspective on the issues of interest in this study. It would also have been interesting to compare the SES of responders and non-responders. However, in a recent review of prospective predictors, mediators, and moderators of NSSI, Valencia-Agudo et al. [34] found that “studies of socioeconomic status (SES), consistently failed to find a relationship with NSSI” (p. 30).

Fifth, we omitted item 2 from the MSI-BPD, which targets NSSI and suicide attempts, replacing it with corresponding questions on the DSHI-9r and about suicide attempts. This might have led to an underestimation of suicide attempts in the study because the question on attempted suicide was asked only if participants endorsed NSSI. However, the MSI-BPD is a screening instrument for BPD and deliberate self-harm is one of the most frequently mentioned symptoms of BPD. It is therefore unlikely that we missed a significant number of people with probable BPD by failing to include individuals with suicide attempts who had never deliberately hurt themselves.

## Conclusions and clinical implications

This study revealed that although NSSI decreases between adolescence and young adulthood, a significant number of individuals continue to report NSSI in young adulthood. Individuals who engaged in NSSI (infrequent, unstable repetitive, or stable repetitive) in adolescence reported a wide range of mental health problems 10 years later. Particularly, individuals with stable repetitive NSSI in adolescence showed substantially increased risk for mental health problems in young adulthood. These findings underscore the need for early identification and treatment of repetitive self-harm to alleviate ill-health in adolescence and reduce the risk of future mental health problems. Moreover, there is a need for further research on designing optimal multifaceted interventions for this purpose. In addition, the present findings suggest that even infrequent and unstable NSSI might be indicators of underlying problems with long-term consequences. The findings also suggest that the detection of such problems might be facilitated if general screening instruments such as the SDQ are complemented by questionnaires such as the DSHI-9r; these latter questionnaires might add important information on adolescents’ dysfunctional ways of relating to themselves, which might serve as a warning sign. Further, an understanding of these problems and their development, and the design of methods of early detection, prevention, and treatment, are important for both the affected individuals and society overall.

We must also emphasize that it is important to obtain a better understanding of what makes many adolescents stop engaging in NSSI. Although a number of cross-sectional and longitudinal studies have explored the intra- and interpersonal factors related to NSSI cessation, such as improved emotion regulation [55, 56] or social support [57–59], none of these studies span beyond 3 years. This means that we have only limited insight into why NSSI prevalence rates decrease from adolescence to young adulthood and what factors predict this decrease. Because cessation of NSSI is a process that often involves relapse [51], a more detailed study of the factors involved in NSSI cessation is important. It is possible that a better understanding of these factors might contribute important information to the development of more efficient interventions.

## Electronic supplementary material

Below is the link to the electronic supplementary material.

Supplementary file1 (DOCX 16 kb)

Supplementary file2 (DOCX 16 kb)

## Footnotes

1 Defined as the child either being born abroad with at least one parent born abroad as well, or being born in Sweden with both parents being born abroad.
