# Supplementary material for: What happens to young adults who have engaged in self-injurious behavior as adolescents? A 10-year follow-up
Source: Eur Child Adolesc Psychiatry. 2020 Apr 21;30(3):475–92. doi: 10.1007/s00787-020-01533-4 (PMC8019412; doi:10.1007/s00787-020-01533-4)
Supplement: Supplementary file 1 — Supplementary file1 (DOCX 16 kb) [file 787_2020_1533_MOESM1_ESM.docx]

Supplementary Table S1.

*Results of Logistic Regression Analysis Predicting Mental Health in Young Adulthood Using the Imputed Dataset (N = 887)*

| NSSI frequency pattern in adolescence as predictor^a^ | b | SE | p | OR |
| --- | --- | --- | --- | --- |
|  |  |  |  |  |
| **On sick-leave longer than 2 months (Yes = 1, No = 0)** | | | |  |
| *Step 1* |  |  |  |  |
| Infrequent NSSI | 0.28 | 0.47 | .54 | 1.33 |
| Unstable repetitive NSSI | 0.72 | 0.55 | .19 | 2.05 |
| Stable repetitive NSSI | 1.63 | 0.48 | <.01 | 5.10 |
| *Step 2* |  |  |  |  |
| Infrequent NSSI | 0.04 | 0.49 | .94 | 1.04 |
| Unstable repetitive NSSI | 0.28 | 0.54 | .60 | 1.32 |
| Stable repetitive NSSI | 0.94 | 0.56 | .10 | 2.56 |
| SDQ total | 0.07 | 0.04 | .09 | 1.07 |
| Gender | 0.58 | 0.42 | .17 | 1.78 |
| **Diagnosed with one or more psychiatric disorders (Yes = 1, No = 0)** | | | | |
| *Step 1* |  |  |  |  |
| Infrequent NSSI | 0.36 | 0.37 | .33 | 1.44 |
| Unstable repetitive NSSI | 0.40 | 0.45 | .38 | 1.49 |
| Stable repetitive NSSI | 1.38 | 0.38 | <.01 | 3.96 |
| *Step 2* |  |  |  |  |
| Infrequent NSSI | 0.15 | 0.38 | .70 | 1.16 |
| Unstable repetitive NSSI | 0.05 | 0.46 | .92 | 1.05 |
| Stable repetitive NSSI | 0.55 | 0.47 | .24 | 1.74 |
| SDQ total | 0.08 | 0.03 | .03 | 1.08 |
| Gender | 1.48 | 0.36 | <.01 | 4.37 |
| **Above cut-off on the McLean screening instrument for BPD (7 or more yes answers = 1, 0–6 yes answers = 0)** | | | | |
| *Step 1* |  |  |  |  |
| Infrequent NSSI | 0.70 | 0.42 | .10 | 2.02 |
| Unstable repetitive NSSI | 0.76 | 0.50 | .13 | 2.13 |
| Stable repetitive NSSI | 1.59 | 0.48 | <.01 | 4.89 |
| *Step 2* |  |  |  |  |
| Infrequent NSSI | 0.52 | 0.45 | .25 | 1.68 |
| Unstable repetitive NSSI | 0.42 | 0.49 | .40 | 1.52 |
| Stable repetitive NSSI | 0.86 | 0.55 | .12 | 2.37 |
| SDQ total | 0.08 | 0.04 | .03 | 1.08 |
| Gender | 0.84 | 0.38 | .03 | 2.31 |

*Note.* NSSI = non-suicidal self-injury. ^a^The original categorical NSSI pattern variable, which had four different values, was recoded into three dichotomous dummy variables with *the No NSSI group* as the reference. Gender: 1 = girl, 0 = boy. Significant coefficients are marked in bold.
