# Supplementary material for: What happens to young adults who have engaged in self-injurious behavior as adolescents? A 10-year follow-up
Source: Eur Child Adolesc Psychiatry. 2020 Apr 21;30(3):475–92. doi: 10.1007/s00787-020-01533-4 (PMC8019412; doi:10.1007/s00787-020-01533-4)
Supplement: Supplementary file 2 — Supplementary file2 (DOCX 16 kb) [file 787_2020_1533_MOESM2_ESM.docx]

|  | Life satisfaction | | | Flourishing | | | Stress | | | Anxiety | | | Depression | | | DSH | | | Emotion dysregulation | | |
| --- | --- | --- | --- | --- | --- | --- | --- | --- | --- | --- | --- | --- | --- | --- | --- | --- | --- | --- | --- | --- | --- |
|  | *b* | *SE* | *p* | *b* | *SE* | *p* | *b* | *SE* | *p* | *b* | *SE* | *p* | *b* | *SE* | *p* | *b* | *SE* | *p* | *b* | *SE* | *p* |
| *Step 1* |  |  |  |  |  |  |  |  |  |  |  |  |  |  |  |  |  |  |  |  |  |
| Infrequent NSSI | -0.27 | 0.72 | .71 | -0.73 | 0.70 | .30 | 1.19 | 0.52 | .02 | 1.04 | 0.38 | .01 | 1.18 | 0.45 | .01 | 0.33 | 0.40 | .41 | 2.68 | 1.31 | .04 |
| Unstable repetitive NSSI | -2.14 | 0.95 | .03 | -1.25 | 0.96 | .19 | 1.86 | 0.59 | <.01 | 1.73 | 0.46 | <.01 | 1.89 | 0.60 | <.01 | 0.60 | 0.50 | .23 | 4.88 | 1.96 | .01 |
| Stable repetitive NSSI | -1.26 | 1.15 | .28 | -2.43 | 1.13 | .03 | 3.23 | 0.70 | <.01 | 2.06 | 0.55 | <.01 | 1.82 | 0.65 | .01 | 3.75 | 0.80 | <.01 | 11.76 | 2.19 | <.01 |
| *R*^2^ | .01 |  |  | .01 |  |  | .05 |  |  | .05 |  |  | .03 |  |  | .06 |  |  | .06 |  |  |
| *Step 2* |  |  |  |  |  |  |  |  |  |  |  |  |  |  |  |  |  |  |  |  |  |
| Infrequent NSSI | 0.42 | 0.84 | .62 | -0.06 | 0.80 | .94 | 0.63 | 0.51 | .21 | 0.70 | 0.37 | .06 | 0.74 | 0.43 | .09 | 0.12 | 0.41 | .77 | 1.43 | 1.49 | .34 |
| Unstable repetitive NSSI | -0.22 | 0.98 | .82 | 0.41 | 0.93 | .66 | 0.77 | 0.59 | .19 | 1.10 | 0.47 | .02 | 0.92 | 0.52 | .08 | 0.33 | 0.53 | .54 | 2.09 | 1.80 | .25 |
| Stable repetitive NSSI | 1.42 | 1.29 | .27 | 0.23 | 1.31 | .86 | 1.27 | 0.80 | .11 | 1.03 | 0.63 | .10 | 0.43 | 0.78 | .58 | 3.15 | 0.89 | <.01 | 6.78 | 2.54 | .01 |
| SDQ total | -0.49 | 0.08 | .01 | -0.45 | 0.08 | .01 | 0.21 | 0.05 | .01 | 0.14 | 0.04 | <.01 | 0.20 | 0.05 | <.01 | 0.07 | 0.05 | .13 | 0.63 | 0.15 | <.01 |
| Gender | 1.32 | 0.64 | .04 | 0.73 | 0.62 | .24 | 1.95 | 0.41 | .01 | 0.45 | 0.31 | .14 | 0.51 | 0.37 | .17 | -.04 | 0.38 | .91 | 3.52 | 1.20 | <.01 |
| *R*^2^ | .09 |  |  | .07 |  |  | .12 |  |  | .08 |  |  | .07 |  |  | .06 |  |  | .11 |  |  |

Supplementary Table S2

*Results of Multiple Regressions Predicting Mental Health In Young Adulthood Using the Imputed Dataset (N = 887)*

*Note.* NSSI = non-suicidal self-injury; The four NSSI patterns were recoded into three dichotomous dummy variables with “No NSSI at T1 and T2” as the reference. Gender is coded as 1 = girl, 0 = boy. Significant coefficients are marked in bold.
